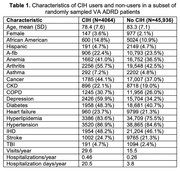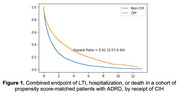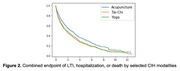# Association of Complementary and Integrative Health Use with Risk of Long‐Term Institutionalization, Hospitalization, or Death in Patients with ADRD

**DOI:** 10.1002/alz.089636

**Published:** 2025-01-09

**Authors:** Yijun Shao, Yan Cheng, Ali Ahmed, Edward Zamrini, Qing Zeng

**Affiliations:** ^1^ George Washington University, Washington, DC USA; ^2^ Washington DC VA Medical Center, Washington, DC USA; ^3^ Irvine Clinical Research, Irvine, CA USA

## Abstract

**Background:**

Complementary and Integrative Health (CIH) encompass many therapeutic modalities including physical, nutritional, psychological, and combination therapies. Small clinical trials on Tai‐Chi, yoga, and acupuncture reported improved cognitive functions. However, there is a knowledge gap regarding effectiveness on long‐term outcomes in patients with Alzheimer’s disease and related dementias (ADRD).

**Method:**

We began by randomly selecting 50,000 patients with ADRD from the Veterans Affairs (VA) electronic health record (EHR) database. We used an NLP tool developed by our team to identify the use of CIH modalities (acupuncture, biofeedback, guided imagery, meditation, Tai‐Chi, yoga) after first ADRD diagnosis. For CIH users, the index date was the date of first CIH use after first ADRD diagnosis. For non‐users, the index date was the encounter date matching the index date of an CIH user with the same ADRD diagnosis year. Then we collected their demographics and clinical data including comorbidities and healthcare utilization from the 5 years before index date.

Next, we matched the non‐users to the CIH users on the propensity scores calculated using the demographics, comorbidities and healthcare utilizations data. On this matched cohort, we performed survival analysis for the combined endpoint of long‐term institutionalization (LTI), hospitalization, or death.

**Result:**

Overall, 4064 (8.1% of 50,000) patients were CIH users. Compared to non‐users, CIH users were younger (78.4 vs. 83.3) with higher proportions of females (3.6% vs. 2.1%) and African Americans (14.8% vs. 10.9%), had higher prevalence in almost all comorbidities, had nearly twice as many visits and hospitalizations per year, and had 4 times longer cumulative hospitalizations during the 5 years before index date (Table 1).

On the propensity score‐matched cohort, the survival analysis yielded a hazard ratio of 0.61 (95% CI: 0.57–0.64) for CIH use (Figure 1). There were some differences between acupuncture users and Tai‐Chi/yoga users (Figure 2).

**Conclusion:**

Our results suggest that CIH treatments in the VA ADRD population are often reserved for patients with more comorbidities and higher healthcare utilizations. In the propensity matched cohort, CIH use is associated with a lower risk in the combined outcome of LTI, hospitalization, or death as the endpoint.